# Somatic gene mutations expose cytoplasmic DNA to co-opt the cGAS/STING/NLRP3 axis in myelodysplastic syndromes

**DOI:** 10.1172/jci.insight.159430

**Published:** 2022-08-08

**Authors:** Amy F. McLemore, Hsin-An Hou, Benjamin S. Meyer, Nghi B. Lam, Grace A. Ward, Amy L. Aldrich, Matthew A. Rodrigues, Alexis Vedder, Ling Zhang, Eric Padron, Nicole D. Vincelette, David A. Sallman, Omar Abdel-Wahab, Alan F. List, Kathy L. McGraw

**Affiliations:** 1 Department of Malignant Hematology, Moffitt Cancer Center & Research Institute, Tampa, Florida, USA.; 2 Department of Internal Medicine, National Taiwan University Hospital (NTUH), Taipei, Taiwan, Republic of China.; 3 Cancer Biology PhD Program, University of South Florida, Tampa, Florida, USA.; 4 Luminex Corporation, Seattle, Washington, USA.; 5 Department of Hematopathology, Moffitt Cancer Center & Research Institute, Tampa, Florida, USA.; 6 Human Oncology and Pathogenesis Program and Leukemia Service, Memorial Sloan Kettering Cancer Center, New York, New York, USA.; 7 Precision Biosciences, Durham, North Carolina, USA.; 8 Laboratory of Receptor Biology and Gene Expression, National Cancer Institute, NIH, Bethesda, Maryland, USA.

**Keywords:** Hematology, Oncology, Leukemias

## Abstract

NLRP3 inflammasome and IFN-stimulated gene (ISG) induction are key biological drivers of ineffective hematopoiesis and inflammation in myelodysplastic syndromes (MDSs). Gene mutations involving mRNA splicing and epigenetic regulatory pathways induce inflammasome activation and myeloid lineage skewing in MDSs through undefined mechanisms. Using immortalized murine hematopoietic stem and progenitor cells harboring these somatic gene mutations and primary MDS BM specimens, we showed accumulation of unresolved R-loops and micronuclei with concurrent activation of the cytosolic sensor cyclic GMP-AMP synthase. Cyclic GMP-AMP synthase/stimulator of IFN genes (cGAS/STING) signaling caused ISG induction, NLRP3 inflammasome activation, and maturation of the effector protease caspase-1. Deregulation of RNA polymerase III drove cytosolic R-loop generation, which upon inhibition, extinguished ISG and inflammasome response. Mechanistically, caspase-1 degraded the master erythroid transcription factor, GATA binding protein 1, provoking anemia and myeloid lineage bias that was reversed by cGAS inhibition in vitro and in *Tet2*^–/–^ hematopoietic stem and progenitor cell–transplanted mice. Together, these data identified a mechanism by which functionally distinct mutations converged upon the cGAS/STING/NLRP3 axis in MDS, directing ISG induction, pyroptosis, and myeloid lineage skewing.

## Graphical abstract

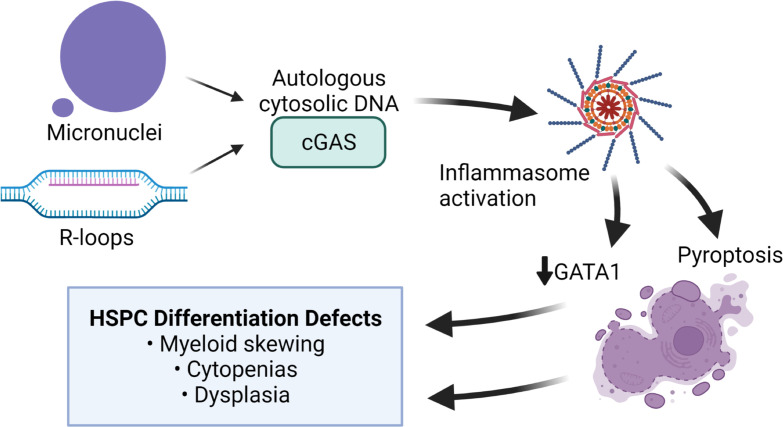

## Introduction

Myelodysplastic syndromes (MDSs) are genetically diverse hematopoietic stem cell malignancies characterized by ineffective and dysplastic hematopoiesis. Recent investigations provide convincing evidence that innate immune activation is a key driver of MDS pathogenesis. Hematopoietic stem and progenitor cells (HSPCs) overexpress TLRs and generate inflammatory cytokines driving medullary expansion of hematopoiesis-inhibitory myeloid-derived suppressor cells (1, 2). Innate immune signaling complexes known as supramolecular organizing centers are constitutively active in MDSs, including the TLR-MyDDosome and NLRP3 inflammasome complexes. These signaling modules induce transcription-dependent and -independent, inflammatory, and lytic cell death responses. NLRP3 inflammasome activation drives both clonal expansion and pyroptotic cell death in MDSs that manifests clinically as cytopenias (3). Both cell-extrinsic and cell-intrinsic circuits appear to induce inflammasome assembly in MDS HSPCs. The myeloid-related S100A9 protein and other danger-associated molecular patterns released upon cytolysis induce canonical inflammasome activation through TLRs in a feed-forward, receptor-driven fashion. Notably, somatic gene mutations (SGMs) involved in mRNA splicing and epigenetic regulation, the most commonly mutated gene classes in MDSs, also evoke NLRP3 inflammasome activation in human and murine models (3). These findings suggest that somatic mutations affecting functionally diverse gene classes may converge upon a common pathway for cell-intrinsic inflammasome activation in MDSs.

One possible mechanism by which SGMs lead to inflammasome activation is through augmented genome instability (4, 5). Somatic mutations evoke replication stress, resulting in transcriptional pauses exposing genomic DNA to cytosolic sensors through accumulation of unresolved R-loops or formation of fragile micronuclei (6, 7). Importantly, HSPC gene expression profiling has shown type I IFNs and IFN-stimulated genes (ISGs) are the most highly expressed genes in MDSs (8), consistent with data sets from mutant-*U2AF1* (National Center for Biotechnology Information’s Gene Expression Omnibus [GEO] GSE66793) and *Tet2*^–/–^ mouse RNA-Seq studies (GEO GSE27816). Sustained type I IFN signaling in murine models results in excess IL-1β elaboration, ineffective hematopoiesis, and BM failure (9). With the exception of TLR4, all other intracellular pattern recognition receptors that induce type I IFNs are nucleic acid sensors activating the IFN regulatory factor 3 (IRF3) or IRF7 transcription factors. Although multiple cytosolic DNA sensors can trigger a type I IFN response, including AIM2-like receptors (ALRs) and family member IFI16, CRISPR editing and murine models show ALRs are dispensable for intracellular DNA IFN response, whereas cyclic GMP-AMP synthase (cGAS) is indispensable (10, 11). cGAS and stimulator of IFN genes (STING) form a cell-intrinsic DNA surveillance pathway that recognizes cytosolic DNA, leading to ISG transcription and NLRP3 inflammasome activation (12, 13). Further, recent data demonstrate that cGAS-induced ISG expression is responsible for defective hematopoiesis in in vivo models (14). cGAS dimerizes upon dsDNA engagement, catalyzing generation of 2′,3′-cyclic GMP-AMP (cGAMP) that binds to and activates STING (12). STING is essential for inflammasome activation in response to cytosolic DNA exposure, promoting activation in 2 ways. First, STING binds NLRP3 in the endoplasmic reticulum, where it anchors the NLR for speck assembly and caspase-1 cleavage (15). Second, STING represses polyubiquitination of NLRP3, which is necessary for inflammasome activation (16).

Here, we investigated sources of cytoplasmic DNA exposure to nucleic acid sensors in primary MDS BM specimens and functionally distinct murine gene mutation models as possible common cell-intrinsic platforms licensing the NLRP3 inflammasome. We report that both RNA splicing and epigenetic modifying gene mutations exposed redundant sources of cytoplasmic DNA to cytosolic cGAS/STING that drove type I IFN response, NLRP3 inflammasome activation, pyroptotic cell death, and caspase-1–directed myeloid lineage skewing, which was rescued by cGAS genetic and pharmacological inhibition. These findings suggest that inhibition of the cGAS/STING/NLRP3 signaling axis may represent a potentially novel therapeutic strategy for investigation in MDSs.

## Results

### SGMs provoke cytosolic DNA exposure.

**Figure 1:**
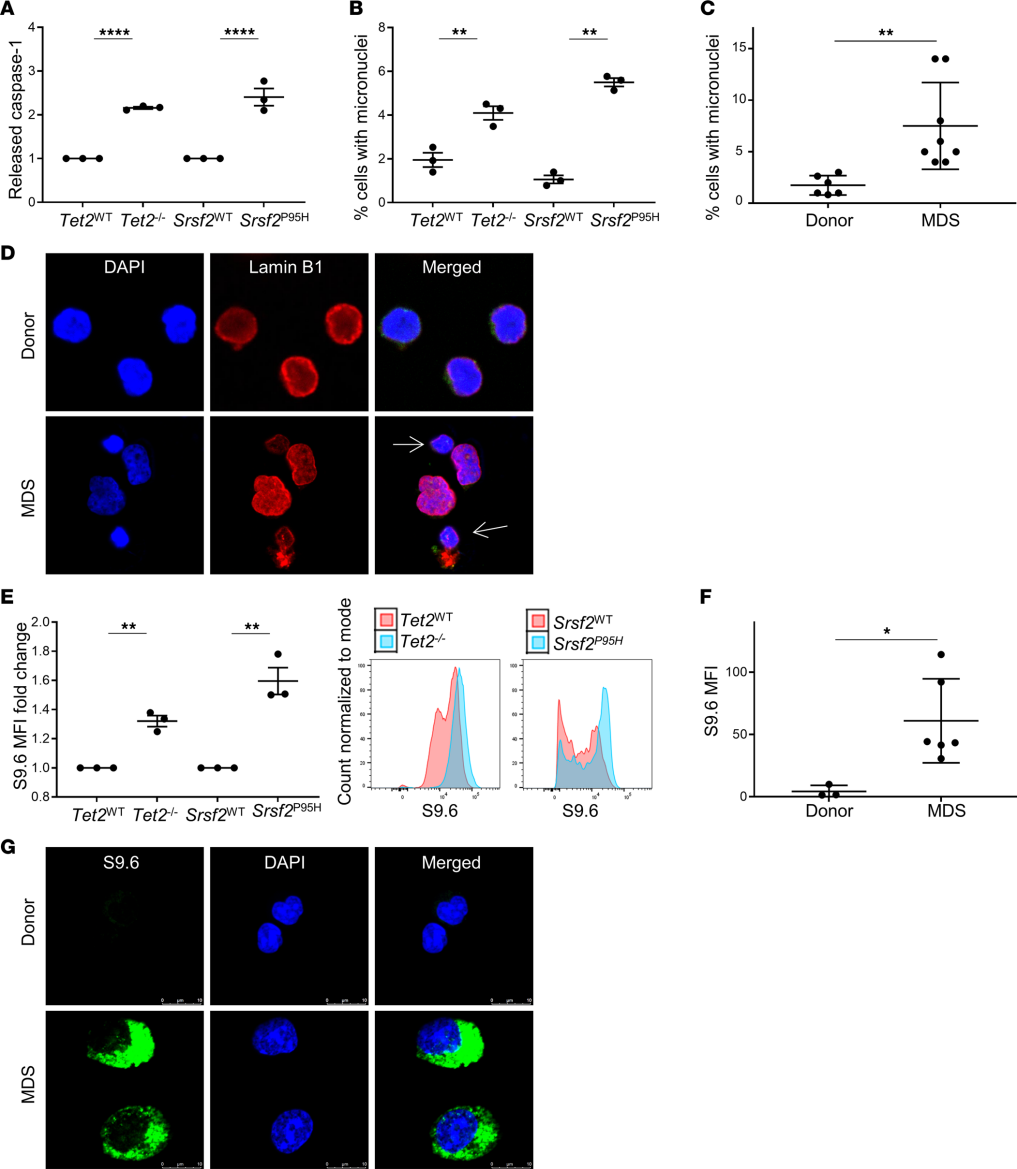
Cytosolic DNA is increased in MDS bone marrow and murine SGM models. (**A**) Released caspase-1 was significantly increased in SGM mutant cell lines compared with WT controls; assessed by Caspase-Glo 1 Inflammasome Assay (*n* = 3 each). (**B**) Percentage of cells with micronuclei was significantly increased in SGM cell lines compared with WT controls (*n* = 3 each), as well as in MDS patient BM-MNCs (*n* = 8) compared with BM donors (*n* = 6) (**C**). (**D**) Representative immunofluorescence images of micronuclei in MDS (*n* = 8) compared with donor BM-MNCs (*n* = 6). (**E**) R-loop expression measured by S9.6 flow cytometry was significantly increased in SGM cell lines compared with WT controls (*n* = 3 each) as well as in MDS BM-MNCs (*n* = 6) compared with controls (*n* = 3): representative histograms. (**F**) Quantification of immunofluorescence images (**G**) of R-loops (shown in green) in MDS (*n* = 6) compared with donor BM-MNCs (*n* = 3); scale bars: 10 μm. Data are presented as mean ± SEM, Student’s *t* test, *P ≤ 0.05, **P ≤ 0.01, ****P ≤ 0.0001.

To investigate the mechanisms by which SGMs evoke inflammasome activation, we first generated renewable cell sources carrying specific mutations common to MDSs by immortalizing BM-HSPCs from *Tet2*^–/–^ and *Srsf2*^P95H^ mutant and respective WT control mice. These common MDS mutations have been demonstrated to induce innate immune activation, leading to proinflammatory cytokine elaboration in MDSs. The immortalized cell lines maintained progenitor phenotypes suitable for investigation and were validated prior to experimentation (Supplemental Figure 1; supplemental material available online with this article; https://doi.org/10.1172/jci.insight.159430DS1) (3, 17, 18). To confirm that these models phenocopy human MDSs, we first evaluated inflammasome activity that we previously demonstrated is a key driver of MDS pathogenesis (3). Both mutant models displayed increased caspase-1 cleavage assessed by the Caspase-Glo 1 assay compared with WT controls, indicative of NLRP3 inflammasome assembly and caspase-1 activation (*P* < 0.0001 for both mutations) (Figure 1A). We next sought to determine whether these mutations caused accumulation of genotoxic stress markers. Micronuclei can be found under conditions of nuclear stress and are formed from lagging mitotic chromosomal DNA and chromatin bridges. We found that both mutant HSPC models displayed a significantly higher mean percentage of micronuclei compared with WT cells (*Tet2*, *P* = 0.0090; *Srsf2*, *P* = 0.0001) (Figure 1B). To determine whether these findings were consistent with human MDSs, we compared primary BM specimens from patients with MDS (*n* = 8) and healthy donors (*n* = 6). Primary MDS specimens displayed a significantly higher fraction of cells containing micronuclei compared with age-matched controls, indicating that the murine mutation variants faithfully recapitulated the findings in human MDS (*P* = 0.0068) (Figure 1C). Impaired micronuclei membrane integrity often leads to membrane collapse and leakage of DNA into the cytosol; therefore, we assessed Lamin B1 staining, which demonstrated micronuclear envelopes were noticeably thinner compared with nuclear membranes, potentially indicative of membrane collapse and DNA compartmentalization compromise (Figure 1D). Additional sources of genomic DNA resolved by replicative stress and transcriptional pauses arising from SGMs can also augment accumulation of RNA:DNA hybrids or R-loops (6). Using the S9.6 antibody that recognizes RNA:DNA hybrids (19), flow cytometry demonstrated a significant increase in cellular R-loops in both of the SGM models studied (*Tet2*, *P* = 0.0011; *Srsf2*, *P* < 0.0030) (Figure 1E). These findings were supported by immunofluorescence in MDS BM mononuclear cells (BM-MNCs) (*n* = 6) compared with healthy donors (*n* = 3) (*P* = 0.0264) (Figure 1, F and G). Of particular interest, although R-loops were visualized in the nuclei, R-loop immunostaining was largely cytoplasmic. Thus, cytosolic DNA sensors may access genomic DNA in MDS progenitors through 2 sources: unresolved R-loops and structurally unstable micronuclei that are induced by both mRNA splicing and epigenetic regulatory gene mutations.

### cGAS is activated by cytoplasmic DNA in MDS.

**Figure 2:**
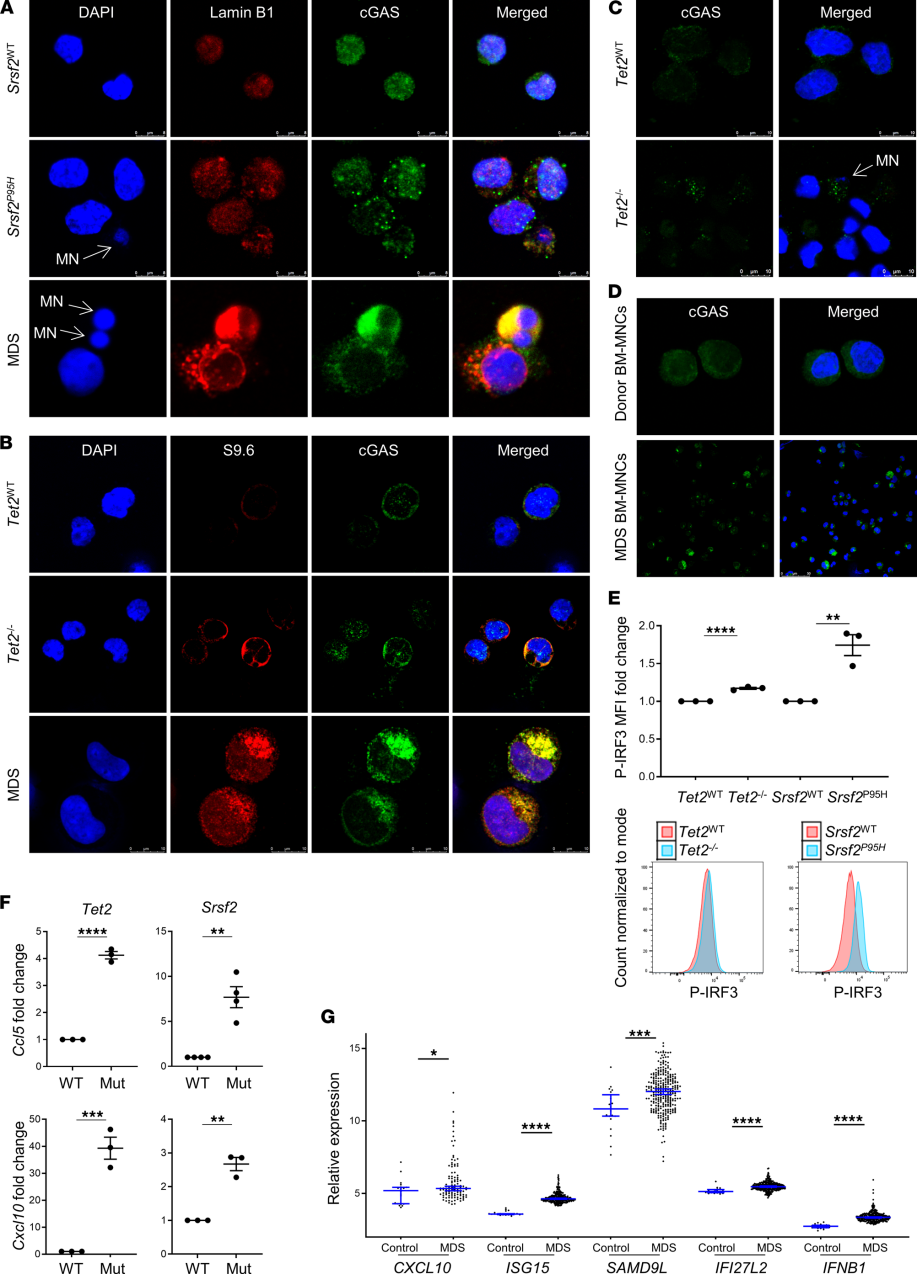
cGAS is activated in SGM models and MDS bone marrow cells. (**A**) Representative immunofluorescence images of SGM mutant cell lines and MDS BM-MNCs with colocalization of cGAS to micronuclei with compromised nuclear membranes. Scale bar: 8 μm. (**B**) Representative immunofluorescence images of colocalization of cGAS and R-loops in SGM mutant cell lines and MDS BM-MNCs. Scale bar: 10 μm. (**C**) Aggregation of cGAS foci was increased in mutant immortalized SGM cells compared with WT controls (cGAS in green, DAPI in blue; original magnification, 3780×). Scale bar: 10 μm. (**D**) Aggregation of cGAS in MDS BM-MNCs (original magnification, 630×) compared with donor BM-MNCs (original magnification, 2940×). Scale bar: 50 μm. (**E**) Phospho-IRF3 was increased in SGM models compared with WT controls; representative flow histograms (*n* = 3 each). (**F**) ISG expression was increased in the immortalized SGM cells compared with WT controls (*n* = 3 each). (**G**) ISG and *IFNB1* expression was significantly increased in MDS BM-MNCs (*n* = 213) compared with age-matched donors (*n* = 20). Data are presented as mean ± SEM. Images are representative of at least 2 independent experiments; Student’s *t* test for in vitro data; Mann-Whitney *U* test for **G**; *P ≤ 0.05, **P ≤ 0.01, ***P ≤ 0.001, ****P ≤ 0.0001.

**Figure 3:**
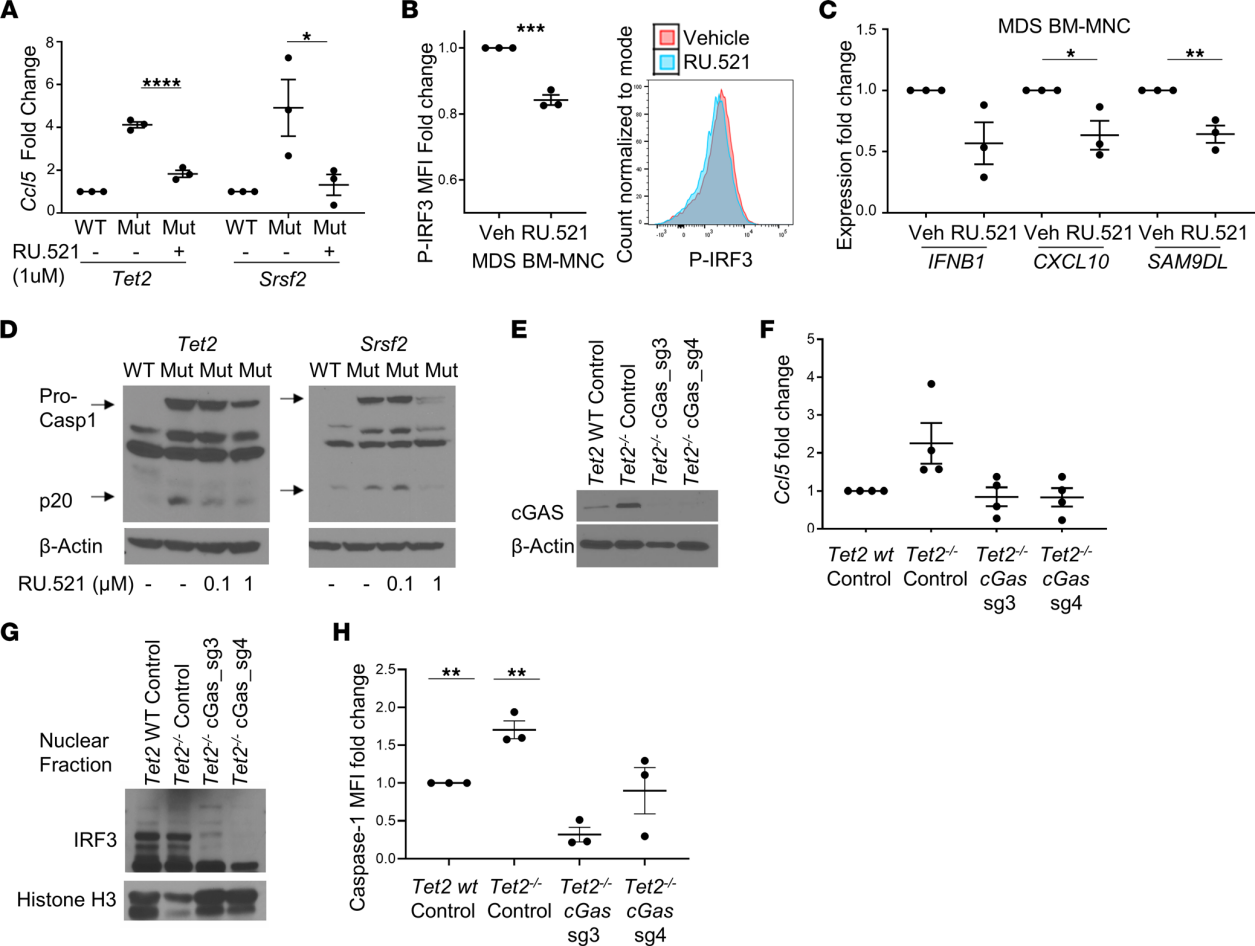
cGAS activation licenses NLRP3 inflammasome activation. (**A**) Treatment with 1 μM of RU.521 for 24 hours decreased *Ccl5* expression nearly to WT levels in both SGM cell lines (*n* = 3 each). (**B**) Phospho-IRF3 assessed by flow cytometry with representative histograms and ISG expression (*n* = 3). (**C**) IFNB1, CXCL10, and SAM9DL expression decreased in low-risk MDS BM-MNCs treated in vitro with 1 μM RU.521 for 48 hours (*n* = 3). (**D**) Cleaved caspase-1 decreased in SGM models with RU.521 treatment. (**E**) cGAS Western blotting of cells treated with scrambled sgRNA (WT and *Tet2*^–/–^ immortalized cells) or cGAS sg_3 or sg_4 (*Tet2*^–/–^). (**F**) CRISPR knockout of cGAS in the *Tet2* SGM cell line decreased *Ccl5* expression (*n* = 4) and nuclear IRF3 (**G**) compared with scrambled control. (**H**) Active caspase-1 assessed by flow cytometry was decreased with cGAS knockdown by CRISPR (*n* = 3). Data are represented as mean ± SEM. Western blots are representative of at least 2 independent experiments. Student’s *t* test; *P ≤ 0.05, **P ≤ 0.01, ***P ≤ 0.001, ****P ≤ 0.0001.

Type I IFNs block maturation of HSPCs, a process that is attenuated by inhibition of the cytosolic DNA sensor cGAS (14). Given the increased prevalence of micronuclei that may expose genomic DNA through porous nuclear membranes and accumulation of cytosolic R-loops in our murine models and primary specimens, we next examined whether cGAS engages these sources of cytoplasmic DNA. We first used immunofluorescence staining to demonstrate cGAS colocalization with both micronuclei (Figure 2A) and cytoplasmic R-loops (Figure 2B) and indeed found that cGAS interacted with both in MDS BM-MNCs as well as the murine mutation variants. Upon engagement with DNA, cGAS dimerizes to catalyze synthesis of the second messenger cGAMP that binds to and activates STING. cGAS engagement of DNA results in the formation of punctate catalytic foci that coalesce into larger spherical droplets by liquid phase transition (20). Accordingly, cGAS foci were observed in murine SGM cells and MDS primary specimens yet limited or absent from WT or normal controls, supporting cGAS dimerization and activation upon DNA engagement (Figure 2, C and D). Upon cGAMP activation, STING translocates from the endoplasmic reticulum to the perinuclear trans-Golgi network, permitting its tetramerization and recruitment of tank-binding kinase 1 (TBK1). TBK1 oligomerizes and autophosphorylates, which enables recruitment and phosphorylation of the transcription factor IRF3, and together with the IKKε kinase, NF-κB. Phospho-IRF3 dimerizes and translocates to the nucleus to direct transcription of IFN-β and ISGs (21). Flow cytometry showed that phosphorylation of IRF3 was significantly increased in both murine SGM variants compared with WT controls (*Tet2*, *P* = 0.0002; *Srsf2*, *P* = 0.0058) (Figure 2E). Accordingly, gene expression of the ISGs *Ccl5* and *Cxcl10* was elevated in the mutant variants compared with WT controls (Figure 2F), consistent with findings in the MDS primary specimens (*CXCL10*, *P* = 0.013; *ISG15*, *P* < 0.0001; *SAMD9L*, *P* = 0.0007; *IFI27L2*, *P* < 0.0001; *IFNB1*, *P* < 0.0001) (Figure 2G). To confirm that the cGAS/STING pathway is responsible for this ISG induction, we treated cells with the cGAS inhibitor RU.521, which selectively suppresses cGAS catalytic activity without affecting retinoic acid–inducible gene 1; TLR-2, -3, -4; or JAK/STAT signaling (22). Indeed, pharmacological inhibition of cGAS significantly reduced *Ccl5* expression in SGM models to levels comparable of that found in WT controls (*Tet2*, *P* = 0.0004; *Srsf2*, *P* = 0.0480) (Figure 3A). These studies were validated in primary MDS specimens where RU.521 treatment significantly reduced IRF3 phosphorylation (Figure 3B), with corresponding suppression of *IFNB1*, *CXCL10*, and *SAMD9L* gene messages in primary MDS specimens (*IFNB1*, *P* = 0.0651; *CXCL10*, *P* = 0.0367; *SAMD9L*, *P* = 0.0073) (Figure 3C). Collectively, these data indicate that cytoplasmic DNA exposed by SGMs engaged and activated a cGAS/STING–type I IFN response in MDS.

### cGAS/STING signaling induces NLRP3 inflammasome activation.

**Figure 4:**
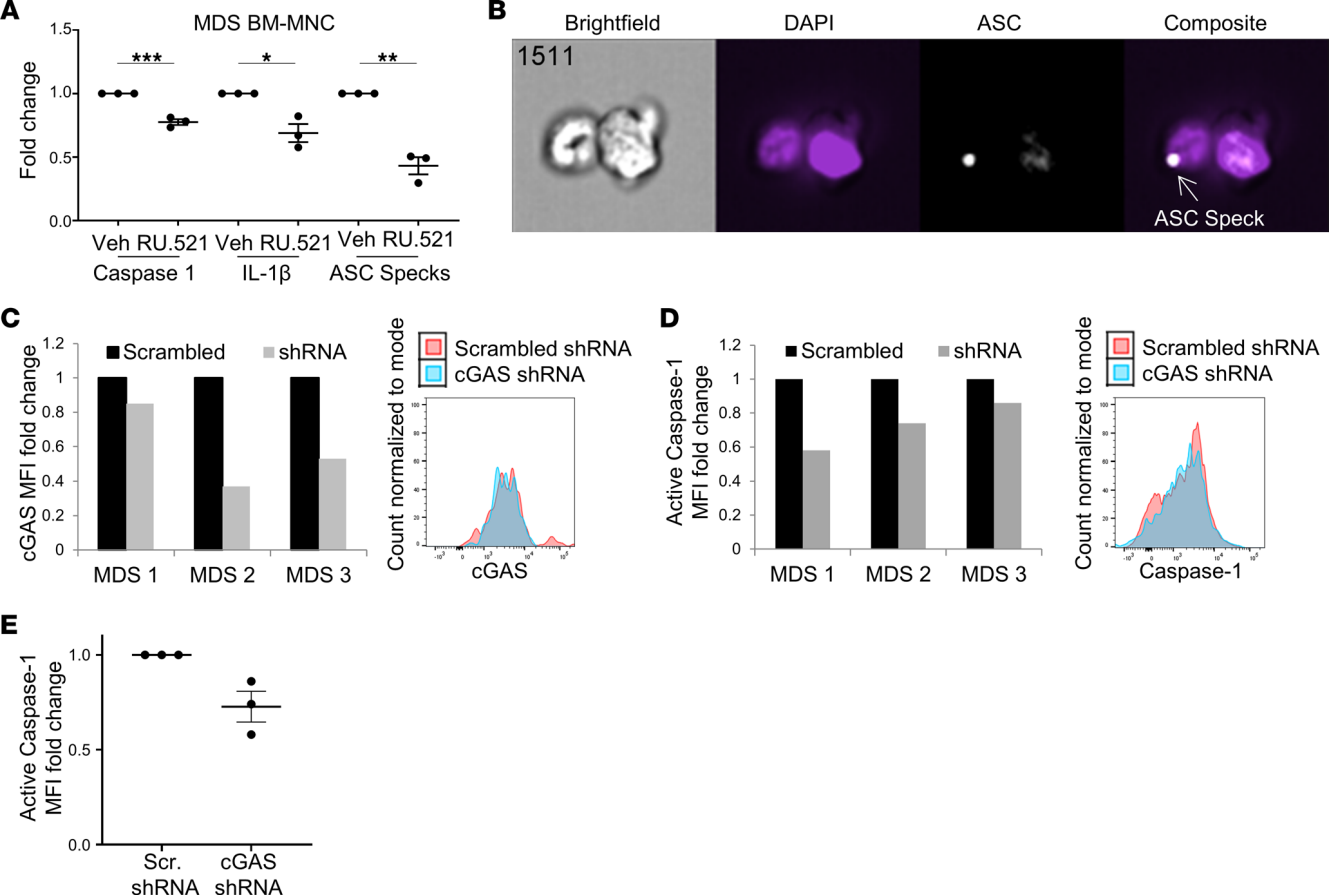
cGAS attenuation inhibits inflammasome activity in primary MDS cells. (**A**) Low-risk MDS BM-MNCs treated in vitro with 1 μM RU.521 for 48 hours (*n* = 3) had decreased active caspase-1, IL-1β, and intracellular ASC specks measured by imaging flow cytometry. (**B**) Representative image of cell with ASC speck. (**C**) Decreased cGAS expression in GFP^+^ primary BM cells with representative flow histogram. (**D**) Decreased caspase-1 activity in 3 MDS BM-MNCs treated with cGAS shRNA with representative flow histogram. (**E**) Average reduction in caspase-1 level of 3 pooled primary MDS specimens treated with shRNA. Data are represented as mean ± SEM. Student’s *t* test; *P ≤ 0.05, **P ≤ 0.01, ***P ≤ 0.001.

Gaidt et al. and others demonstrated that the cGAS/STING pathway is essential for NLRP3 inflammasome activation in response to cytosolic DNA exposure in myeloid cells (13, 23). From the trans-Golgi network, STING transitions to the lysosome, where it permeabilizes the lysosomal membrane, triggering cathepsin B and Ca^2+^ ion release with the consequent activation of the calcium/calmodulin-dependent protein kinase II/TAK1/JNK pathway that activates the NLRP3 inflammasome (23). To determine whether cGAS/STING functions as the principal cell-autonomous pathway provoking inflammasome activation by SGMs, we treated the murine SGM HSPC variants with the cGAS inhibitor RU.521. Incubation of SGM models with RU.521 reduced caspase-1 cleavage in a concentration-dependent manner (Figure 3D), demonstrating cGAS/STING regulated NLRP3 inflammasome activation by SGMs. Moreover, total levels of pro–caspase-1 were concordantly reduced, consistent with the known transcriptional priming of NLRP3 inflammasome components by type I IFNs (13). To confirm that these findings were not due to nonspecific effects of pharmacological perturbation, we used CRISPR gene editing to knock out *cGAS* expression in the immortalized *Tet2*^–/–^ HSPCs. We used 2 cGAS guide RNAs (gRNAs) (sg_3 and sg_4) in the *Tet2*^–/–^ cells and compared and compared data from cGAS gRNA with transduction of a scrambled gRNA in WT and *Tet2*^–/–^ cells. Cells were transduced with CRISPR plasmid containing lentivirus and expanded after GFP sorting. Indeed, protein expression of cGAS was nearly completely abolished using both sg_3 and sg_4 (Figure 3E). Similar to pharmacological inhibition, cGAS knockdown by gene editing resulted in a decrease in ISG expression, returning *Ccl5* mRNA expression in *Tet2*^–/–^ cells to levels commensurate with WT cells (Figure 3F). Decreased ISG expression was accompanied by a corresponding reduction in IRF3 activity demonstrated by decreased nuclear localization of the transcription factor (Figure 3G). Importantly, CRISPR knockdown of *cGAS* also suppressed caspase-1 activity, further indicating that cGAS/STING directs inflammasome activation in response to cytoplasmic DNA sources exposed by SGMs (Figure 3H). These findings were then validated by treatment of primary MDS BM-MNCs with RU.521, which suppressed inflammasome activity as evidenced by a reduction in caspase-1 cleavage, IL-1β maturation, and generation of adaptor protein apoptosis-associated speck-like protein containing CARD (ASC) specks, a reliable surrogate for inflammasome activity (24) (Figure 4, A and B). Last, knockdown of *cGAS* with shRNA in 3 primary MDS specimens consistently showed a marked reduction in caspase-1 cleavage (Figure 4, C–E), confirming that cGAS engagement of DNA licensed NLRP3 inflammasome activation in MDS.

### Cytosolic R-loops are evoked by RNA polymerase III.

**Figure 5:**
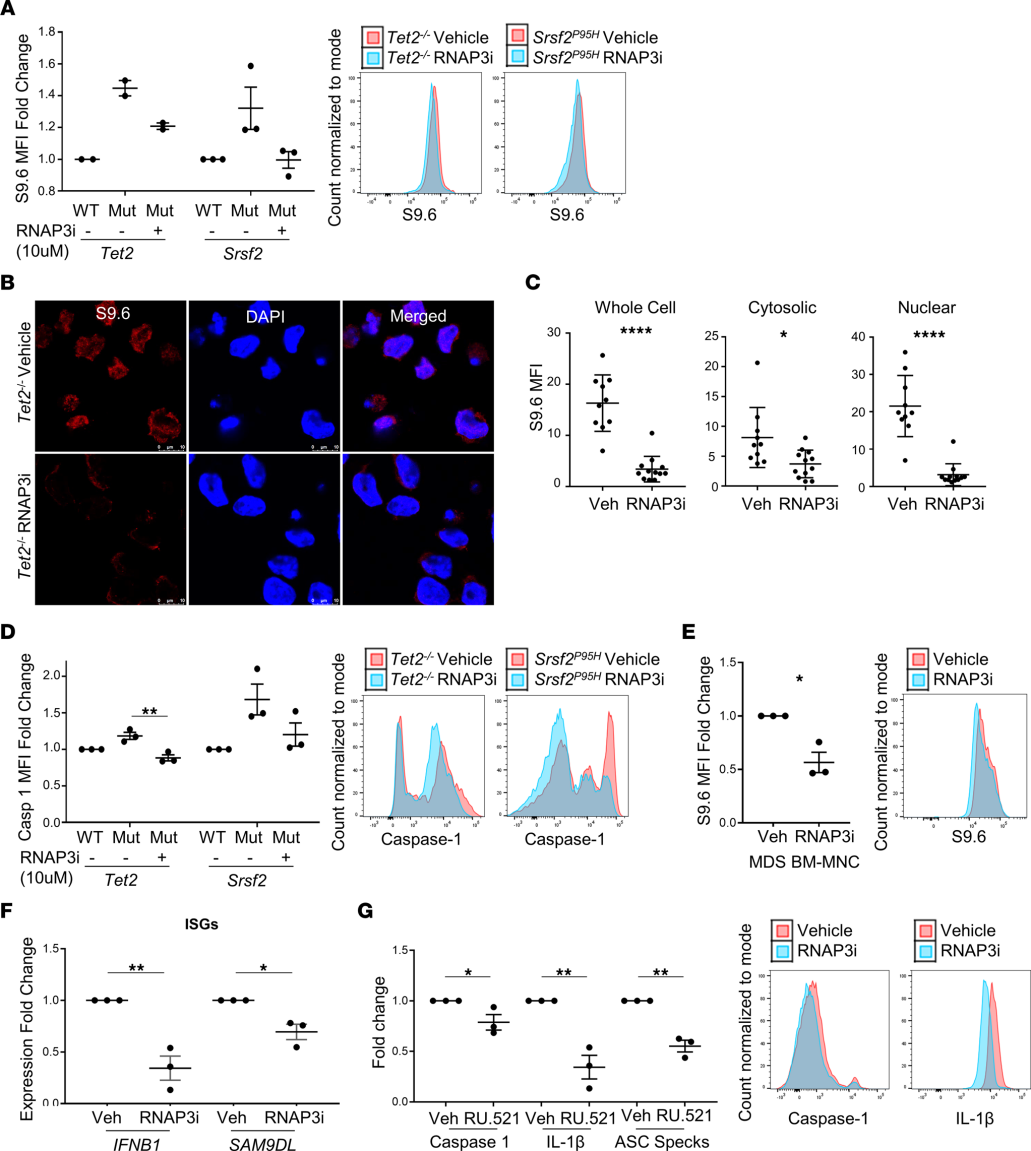
RNAP3 inhibition suppresses R-loop accumulation and inflammasome activity. (**A**) SGM models were treated with ML-60218 (RNAP3 inhibitor, RNAP3i) for 72 hours, and R-loop (S9.6) expression was assessed by flow cytometry (*n* = 2 each for *Tet2* cells and *n* = 3 each for *Srsf2* cells) with representative flow histograms. (**B**) Representative IF images of R-loop reduction in *Tet2*^–/–^ immortalized cells treated with RNAP3i (scale bar: 10 μm) and (**C**) MFI quantitation of immunofluorescence (*n* = 10 vehicle-treated cells and *n* = 12 RNAP3i-treated cells). (**D**) Caspase-1 activity by flow cytometry with representative histograms (*n* = 3 each). (**E**) Low-risk MDS BM-MNCs treated in vitro with 10 μM RNAP3i for 72 hours (*n* = 3), resulting in a decrease in R-loops with representative histogram, ISGs (*n* = 3 each) (**F**), and inflammasome markers (caspase-1, IL-1β, and ASC specks (*n* = 3 each) (**G**), with representative flow histograms. Data are presented as mean ± SEM. Student’s *t* test; *P ≤ 0.05, **P ≤ 0.01, ****P ≤ 0.0001.

Nuclear RNA:DNA hybrids are transcribed largely by RNA polymerase II (25); however, recent investigations have shown that RNA:DNA hybrids are also demonstrable in the cytosol of human tumor cells of varied histogenic origin. These cytosolic R-loops are generated by RNA polymerase III (RNAP3) and exported to the cytosol by the nuclear pore transport protein exportin-1 (26). RNAP3 transcribes 5S rRNA, tRNA, certain retroelements, and noncoding RNAs, and is regulated by oncogenes and tumor suppressor genes. In one study, inhibition of RNAP3 and not DNA polymerases was sufficient to abolish cytosolic RNA:DNA hybrid generation, indicating that RNAP3 transcripts may be the source of cytosolic RNA:DNA hybrids recognized by cGAS (26). To determine whether the excess accumulation of cytosolic R-loops induced by SGMs is transcribed by RNAP3 and whether they are sufficient to license cGAS-induced inflammasome oligomerization, we first investigated RNAP3 gene expression. Microarray expression of each RNAP3 subunit was found to be significantly elevated in MDS specimens compared with controls (Supplemental Table 1). To determine whether RNAP3 is responsible for the accumulation of R-loops in SGM variants and MDS cases, we incubated cells with the highly specific RNAP3 inhibitor ML-60218 and assessed changes in R-loop accumulation and inflammasome activity. ML-60218 treatment of the immortalized SGM HSPCs markedly suppressed cellular R-loops assessed by flow cytometry (Figure 5A) and immunofluorescence (Figure 5, B and C). Accordingly, we found that R-loop suppression by ML-60218 abolished caspase-1 cleavage, indicating that RNA:DNA hybrids generated by RNAP3 may be the predominant source of cytosolic DNA inducing DNA-directed inflammasome activation by SGMs (Figure 5D). We next treated primary MDS specimens (*n* = 3) with ML-60218, which significantly reduced R-loops (*P* = 0.0381) (Figure 5E), in addition to *IFNB1* and *SAMD9L*, but not *CXCL10* expression (*IFNB1*, *P* = 0.0050; *SAMD9L*, *P* = 0.0150) (Figure 5F). Treatment also significantly reduced active caspase-1, IL-1β, and ASC specks (*P* = 0.0162, 0.0050, 0.0016, respectively) (Figure 5G), indicating that RNAP3 inhibition attenuated inflammasome activity in primary MDS specimens. Overall, these data indicate that SGM deregulated RNAP3 in MDS, resulting in excess cytoplasmic R-loop generation that was engaged by cGAS/STING.

### cGAS/STING/NLRP3 signaling directs myeloid lineage bias by SGMs.

**Figure 6:**
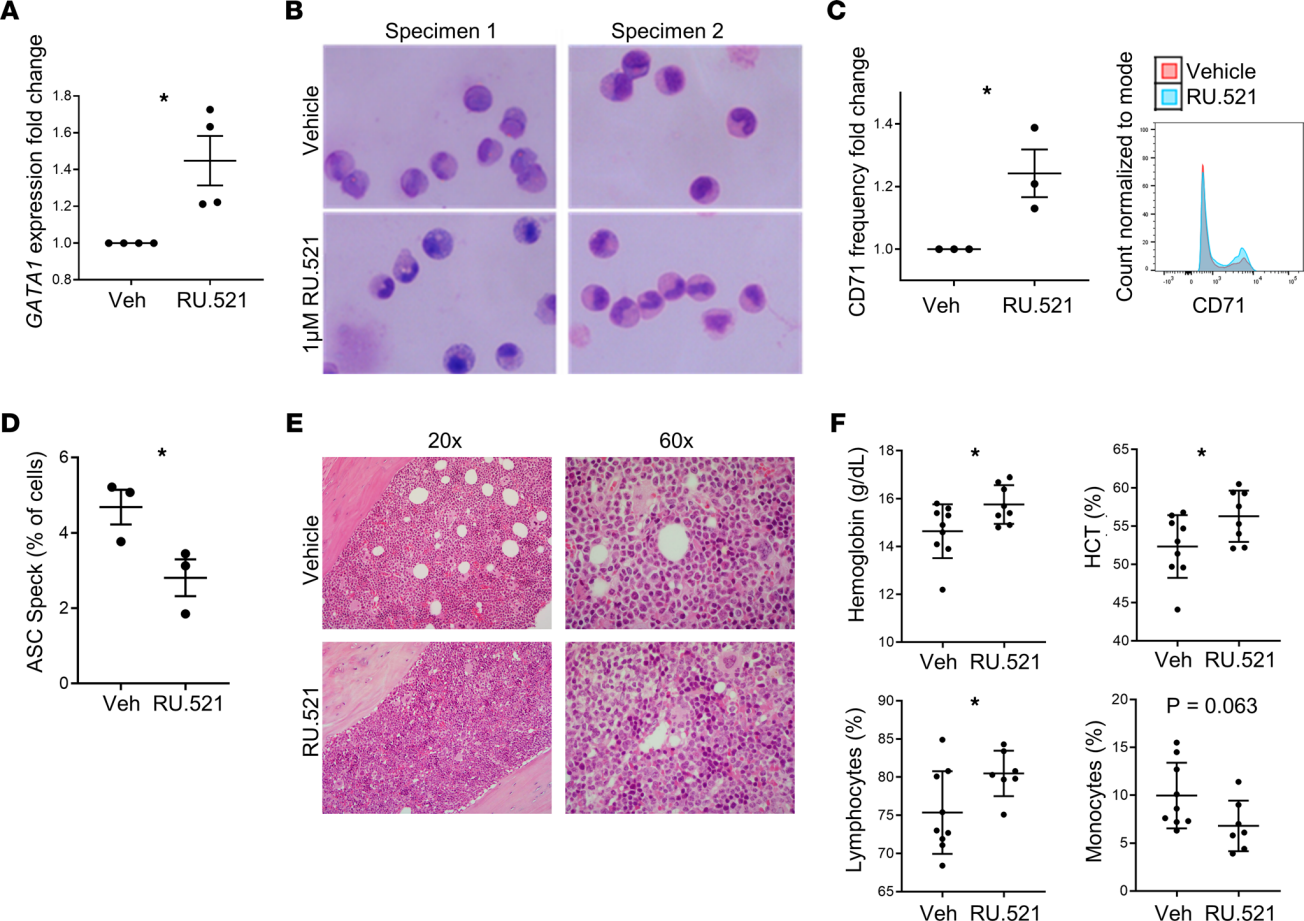
Treatment with cGAS inhibitor restores erythroid differentiation. (**A**) Treatment with the cGAS inhibitor increased *GATA1* transcription (*n* = 4) in MDS BM-MNCs, differentiation evidenced by Wright-Giemsa staining (*n* = 2) (**B**), and CD71 expression (*n* = 3); representative flow histogram (**C**) in MDS BM-MNCs. The original image was taken with a 40× objective on EVOS FL Auto microscope. (**D**) BM-MNC ASC specks decreased in *Tet2*^–/–^ CD45.2 BM-MNCs treated in vivo for 6 weeks with RU.521 compared with vehicle (*n* = 3, each); (**E**) increased erythroid islands in the bone marrow from mice treated with RU.521compared with mice treated with vehicle; and (**F**) increased hemoglobin, hematocrit, and lymphocytes and decreased monocytes in treated mice (*n* = 8) compared with vehicle (*n* = 9). Data are presented as mean ± SEM. Student’s *t* test; *P ≤ 0.05.

The mRNA splicing and epigenetic modifying gene mutations common to MDSs promote granulo-monocytic skewing at the expense of erythroid differentiation (27–30). Erythroid versus myeloid lineage fate is governed by the balance of the GATA binding protein 1 (GATA1) and the *Spi1*-encoded PU.1 transcription factors, respectively, that physically interact and mutually repress respective target genes (31). Caspase-1 regulates erythroid and myeloid lineage bias through degradation of the master erythroid transcription factor, GATA1, thereby raising the PU.1/GATA1 ratio to favor myeloid commitment (32). To determine whether inflammasome activation by cGAS drives myeloid lineage skewing, we treated HSPCs with the cGAS inhibitor RU.521. Treatment of primary MDS specimens resulted in marked upregulation of *GATA1* (*P* = 0.0159) (Figure 6A), accompanied by expansion of erythroid precursors (Figure 6B) and increased expression of the CD71 transferrin receptor (*P* = 0.0338) (Figure 6C), indicating reversal of myeloid lineage skewing. To confirm the functional role of the cGAS/STING/NLRP3 axis in vivo, we transplanted *Tet2^fl/fl^* CD45.2 donor BM-MNCs into CD45.1 recipient mice and began daily treatment with RU.521 (10 mg/kg by i.p. injection) at 14 weeks. After 6 weeks of treatment with the cGAS inhibitor, the ASC speck percentage of CD45.2 cells was significantly reduced, with a corresponding increase in the erythroid compartment demonstrated by H&E staining of murine femurs (Figure 6, D and E). Further, cGAS inhibition caused statistically significant increases in hemoglobin (*P* = 0.0323) and hematocrit (*P* = 0.0453) accompanied by a reduction in monocytes (*P* = 0.0626) and a rise in lymphocytes (*P* = 0.0319) (Figure 6F). These data indicate that myeloid lineage skewing by SGMs arose from activation of the cGAS/STING/NLRP3 axis that directed caspase-1–mediated degradation of GATA1.

## Discussion

**Figure 7:**
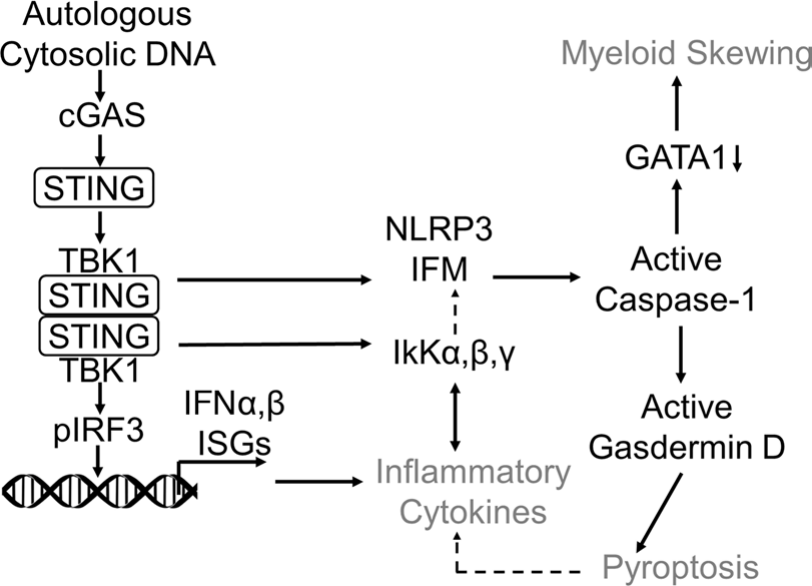
Autologous cytoplasmic DNA activates the cytoplasmic sensor, cGAS, directing inflammasome activation and differentiation defects in MDS. Schematic summarizing cGAS activation as a result of somatic gene mutations that cause accumulation of autologous cytoplasmic DNA and activate cGAS; subsequent inflammasome activity leads to proinflammatory cytokine elaboration, pyroptotic cell death, and HSPC differentiation defects. IFM, inflammasome.

Somatic mutations involving mRNA splicing or epigenetic regulatory genes are the most common genetic abnormalities in MDSs. Although frequently co-occurring, individual gene mutations from either of these functionally distinct classes are singularly sufficient to induce NLRP3 inflammasome activation, which drives ineffective hematopoiesis through pyroptotic cell death (3). To our knowledge, our investigations demonstrate for the first time that these mutations converge upon the cGAS/STING/NLRP3 signaling axis as a common pathway directing inflammasome activation in MDSs (Figure 7). We showed that the cytosolic DNA sensor cGAS engaged unresolved R-loops and structurally unstable micronuclei in murine mutation models and MDS HSPCs. cGAS/STING activation led to transcription factor IRF3 activation directing type I IFN response and ISG transcription. cGAS genetic or pharmacological inactivation extinguished ISG expression and caspase-1 cleavage, indicating that this signaling axis is singularly responsible for IFN and inflammasome induction. Particularly important, licensing of cGAS/STING/NLRP3 by exposed cytoplasmic DNA provides a mechanistic rationale for inflammasome induction in age-related clonal hematopoiesis, which accelerates atherosclerotic plaque formation and cardiovascular risk (33, 34). Although other nucleic acid sensors recognizing dsRNA might also detect R-loops, these receptors exhibit a preference for non-self RNA or are localized in endolysosomal compartments, where they are shielded from endogenous RNA. These findings also provide a mechanistic rationale for findings by Zhou et al. that accumulation of cytosolic dsDNA in senescent cells triggers the induction of pyroptosis (35). Although we found that cGAS engages both micronuclei and R-loops, the latter appear to be the principal source of DNA driving cGAS/STING/NLRP3 signaling. While R-loops were demonstrable in the nuclei of mutant and MDS HSPCs, they were abundantly cytosolic, permitting cGAS engagement. Surprisingly, cytosolic R-loops were largely generated by RNAP3, evidenced by R-loop depletion and extinction of ISG and inflammasome response upon RNAP3 inhibition.

Murine models have shown that mRNA splicing and epigenetic modifying gene mutations promote myeloid lineage skewing at the expense of erythroid differentiation (27–30). Here, we report that myeloid lineage skewing induced by SGMs arose from caspase-1–mediated degradation of GATA1. The balance between GATA1 and the myeloid transcription factor PU.1 governs lineage commitment; therefore, degradation of GATA1 favors myeloid skewing (32, 36). Indeed, MDS HSPC incubation with a potent cGAS inhibitor stabilized GATA1 and induced erythroid expansion. In a *Tet2*-deficient mouse HSPC transplant model, we found cGAS inhibition restored hemoglobin production, resolved anemia, and suppressed monocyte production. Our finding that myeloid lineage skewing by somatic mutations arose from caspase-1 degradation of GATA1 enabled by the cGAS/STING/NLRP3 axis provides mechanistic rationale for the recent report that oncogenic *Kras*^G12D^ induces myeloproliferation as a result of NLRP3 inflammasome activation (37).

Recent reports have shown that MDS HSPCs harboring SGMs activate noncanonical NF-κB signaling to confer a competitive advantage in the inflammatory MDS microenvironment (38). This inflammation-directed activation of alternative NF-κB signaling was dependent upon upregulation of the ubiquitin-modifying enzyme A20 encoded by the ISG *TNFAIP3*. Through its binding to and dissociation of the NF-κB–inducing kinase (NIK) polyubiquitination complex, A20 provokes degradation of TNF receptor–associated factor 2 (TRAF2) and TRAF3 to stabilize NIK (39). Activation of the cGAS/STING/IRF3 axis triggers degradation of TRAF3, thereby stabilizing NIK and activating the noncanonical NF-κB signaling pathway (40). Upon autophosphorylation, NIK oligomerizes and associates with the STING/TBK1/IRF3 complex. NIK binding suppresses decoration of STING with K48-linked ubiquitin chains, enhancing stability and strengthening type I IFN response. Weinreb et al. recently reported that excess R-loops engage cGAS/STING to trigger an inflammatory cascade that directs an increase in HSPC production, thereby endowing a self-renewal benefit to mutant HSPCs (41). Importantly, the data here provide a mechanistic rationale for the concurrent induction of NLRP3 inflammasome–enabled caspase-1 activation, myeloid skewing, and the competitive renewal advantage of clonal HSPCs in the inflammatory MDS microenvironment. Together, these investigations suggest that inhibition of the cGAS/STING/NLRP3 signaling axis may represent a promising therapeutic strategy for investigation in MDSs.

## Methods

### Cells.

BM-MNCs harvested from *Tet2*^–/–^ and *Srsf2*^P95H^ mutant mice and their respective WT controls were immortalized for use as MDS SGM cell line models (42, 43). Murine cells were immortalized using a conditional *Hoxb8* transgene as previously described (44). Briefly, BM-MNCs were harvested from mice and transduced with an estrogen-regulated, HoxB8-containing retrovirus and cultured in RPMI (Gibco, Thermo Fisher Scientific) supplemented with 10% FBS, 1% penicillin-streptomycin, 50 ng/mL recombinant murine stem cell factor, and 0.5 μM β-estradiol. Primary BM-MNCs were isolated using Ficoll-Paque (GE Healthcare) from patients with MDS and healthy donors who provided informed consent at the Moffitt Cancer Center or NTUH or were purchased from AllCells. Primary BM-MNCs were cultured in RPMI supplemented with 10% autologous plasma. Cells were treated with the cGAS inhibitor RU.521 (Aobious) or RNAP3 inhibitor ML-60218 (MilliporeSigma) at 1 μM or 10 μM, respectively, for 24–72 hours.

### Dot blotting.

DNA was extracted using QIAamp DNA Mini Kit (QIAGEN) following the manufacturer’s protocol and denatured for 5 minutes at 99°C and then placed on ice for 10 minutes. DNA was neutralized by adding 1 volume of 2 M ammonium sulfate pH 7.0 and incubating on ice for 5 minutes. Samples were then sonicated for 30 seconds, quantitated, and diluted to equal concentrations. Next, 2 μg of DNA was dotted on the membrane and allowed to fully dry. Membranes were then cross-linked with UV, blocked in 10% nonfat dry milk for 15–20 minutes, and incubated in 5-hmC primary antibody (Active Motif, 1:1000, catalog 39069) for 15 minutes. The membrane was then washed 3 times at room temperature with PBS–Tween 20 (PBST) and incubated in 1:5000 secondary antibody for 15 minutes at room temperature. The membrane was washed and developed using chemiluminescence. The membrane was then washed in 0.4 μg/mL of propidium iodide, washed with PBS, and imaged.

### PCR sequencing for cell line validation.

*SRSF2^P95H^* mutation was confirmed by PCR amplification of cDNA using forward primer 5′ ACGTGTACATTCCGCGGGAC 3′ and reverse primer 5′ ATCTGGAGACGGAGGAGGAC 3′ with the following amplification: 94°C for 2 minutes, 35 cycles of 94°C for 30 seconds, 55°C for 30 seconds, 72°C for 30 seconds, and a final extension at 72°C for 5 minutes.

### Oligonucleotides and antibodies.

All quantitative PCR primers and sgRNAs are listed in Supplemental Table 2, and all antibodies are listed in Supplemental Table 3.

### Western blotting.

Cells were harvested and washed 3 times in PBS and then lysed using 1× RIPA buffer containing 250 μM Na_3_VO_4_, 2 μg/mL aprotinin, 2 μg/mL leupeptin, 0.2 μg/mL pepstatin A (all 3 from MilliporeSigma), and 500 μM PMSF. Proteins were separated by SDS-PAGE and transferred onto PVDF membranes. Membranes were blocked with 5% nonfat dry milk in PBST and then incubated with indicated antibodies. Membranes were developed using ECL or ECL+ per the manufacturer’s protocol (Pierce, Thermo Fisher Scientific).

### Active Caspase-Glo 1 Inflammasome Assay.

Cell culture supernatants were used to measure active caspase-1 using the Caspase-Glo 1 Inflammasome Assay (Promega) per the manufacturer’s protocol.

### CRISPR/Cas9 gene editing.

cGAS genes were deleted by CRISPR/Cas9 gene editing by inserting 1 of 2 *cGAS* or 1 scrambled gRNA into a GFP-expressing pL-CRISPR.SFFV.GFP plasmid (pL-CRISPR.SFFV.GFP that was a gift from Benjamin Ebert, Dana-Farber Cancer Institute, Boston, Massachusetts, USA) (Addgene plasmid 57827) (45). The plasmid was digested using BsmBI and gel purified. Forward and reverse guide oligonucleotides were purchased from Integrated DNA Technologies. CACC on forward and AAAC on reverse oligonucleotides were added 5′ for plasmid ligation. Guide-containing plasmids were transformed into Stbl3 competent cells. CRISPR plasmids were packaged into lentivirus using HEK293T cells transfected by incubating 2600 ng of shRNA plasmid, 30 μL Lipofectamine 2000 (Invitrogen), and 26 μL MISSION Lentiviral Packaging Mix (Sigma-Aldrich) in 500 μL of Opti-MEM I (Life Technologies) for 15 minutes at room temperature. This mixture was then added to 70% confluent HEK293T cells (ATCC) with 4 mL serum-free Opti-MEM in a 100 mm dish. Virus-containing supernatants were collected 48 hours and 72 hours after transfection. Lentiviral transduction of immortalized murine cells was performed by spinfection (2000*g* for 2 hours) method (1 mL virus with titer of at least 10^5^ infectious units/mL added to 500 μL containing 250,000 cells per well in a 24-well plate) with 8 μg/mL polybrene. After spinfection, cells were incubated at 37°C in a humidified incubator (5% CO_2_) for 1 hour, and then 3 mL of complete media (RPMI with 10% FBS, 1% penicillin-streptomycin, 50 ng/mL recombinant murine stem cell factor, and 0.5 μM β-estradiol) was added. After 3 days, GFP^+^ cells were sorted and expanded. Cells were cultured in complete media and harvested for downstream assays.

### Immunofluorescence microscopy.

Cytospins were fixed with Cytofix (Becton Dickinson) for 10 minutes at 37°C, washed with 0.1 M glycine (Thermo Fisher Scientific) and PBS, and permeabilized/blocked with 0.2% Triton X-100/2% BSA for 30 minutes at room temperature. Primary antibody staining was performed for 1 hour at room temperature, then washed 3 times with PBS, then incubated in secondary antibody for 1 hour at room temperature. Cells were mounted with ProLong Gold Antifade Mountant with DAPI (Invitrogen) and imaged using a Leica SP8 confocal microscope. At least 100 cells were assessed for each replicate.

### Quantitation of ASC specks.

Primary BM-MNCs were fixed with BD Cytofix for 10 minutes, washed with 0.1 M glycine, permeabilized/blocked with 0.2% Triton X-100/2% normal mouse serum (Abcam) for 1 hour, stained with mAb ASC conjugated to Alexa Fluor 647 (Santa Cruz Biotechnology catalog sc-514414) for 1 hour, and resuspended in 1 μg/mL DAPI (Sigma-Aldrich). Data were acquired with an Amnis ImageStream Mark II imaging flow cytometer and analyzed using IDEAS (Luminex Corporation). Data were analyzed as previously described (46) with modifications. Samples were run on the lowest speed, and at least 3000 images were collected with 60× objective. A masking strategy was applied to remove doublets, identify ASC speck–containing cells, and differentiate specks from diffuse staining.

### Murine studies.

BM-MNCs were harvested from 3- to 5-week-old *Tet2^fl/fl^* donor mice (The Jackson Laboratory, stock 017573). CD45.1 recipient mice (stock 002014) were sublethally irradiated with 6 Gy for 24 hours prior to transplant. At transplant, donor cells were thawed and washed with 10 mL IMDM. Cells were then treated with 0.1 mg/mL DNase for 15 minutes at room temperature. One million cells in 100 μL saline were tail vein injected per mouse. Mice were induced with 250 μg pI:pC twice every other day starting 7 days after injection. Mice with at least 70% CD45.2 repopulation were used. Mice were treated with vehicle (5% 1-Methyl-2-pyrrolidinone; 5% Solutol, both from MilliporeSigma; 90% saline) or 10 mg/kg RU.521 by i.p. injection once a day, 7 days a week, for 6 weeks at 14 weeks after induction. Tissues were fixed in formalin for 48 hours, then preserved in 70% ethanol until analysis.

### Gene expression analysis.

RNA was extracted by QIAGEN RNeasy Mini Kit and reverse-transcribed using qScript XLT cDNA Supermix (Quantabio). *IFNB1/Ifnb1* and *GATA1* were quantified using TaqMan Advanced Gene Expression Assays, TaqMan Fast Advanced Master Mix, and 7900HT Fast Real-Time PCR System (Life Technologies). *ISG15/Isg15*, *CXCL10/Cxcl10*, *SAMD9L*, and *Ccl5* were quantified with primers listed in Supplemental Table 2 and SYBR Green PCR Master Mix (Life Technologies). Ct values were normalized to *ACTB*/*Actb* for TaqMan and *GAPDH/Gapdh* for SYBR assays; the 2-ΔΔCt method was used to calculate fold-change. Gene expression profiling was also obtained from 213 patients with MDS and 20 healthy donors from the NTUH using HumanHT-12 v4 Expression BeadChip (Illumina) (47).

### shRNA.

cGAS shRNA constructs were purchased from Origene (TL305813) and packaged into lentivirus as previously described (3). shRNAs were used either as single shRNAs (shRNA A, B, C, or D) or pooled (equal concentrations of shRNA A–D), and for the analysis, conditions with the greatest reduction in expression of cGAS were assessed by flow cytometry, which varied among cases.

### Flow cytometry.

For active caspase-1 and caspase-3/7 staining, cells were washed with PBS/2% FBS and incubated with either FAM-FLICA or FLICA 660 (ImmunoChemistry Technologies) per the manufacturer’s protocol. For fixable viability staining, cells were washed; stained with Zombie NIR, violet, or ultraviolet (BioLegend) for 15 minutes at 1:1000 dilution at room temperature; then washed with PBS/2% FBS. For antibody staining, cells were incubated with human or mouse Fc block (Miltenyi Biotec) and stained 20 minutes at 4°C. Data were acquired using a BD Symphony, Canto, or LSRII flow cytometer (Becton Dickinson). FCS files were analyzed using FlowJo v10 software (Tree Star). Relative MFIs between replicates were compared as fold-change differences rather than raw MFI values because of variation between flow cytometers.

### Statistics.

A 2-tailed Student’s *t* test was used to compare means for in vitro and in vivo experiments. Statistical analyses were performed using GraphPad Prism. A Mann-Whitney *U* test was used to compare median expression of ISGs in MDS patient specimens compared with BM donors. Statistical analyses were performed by using SPSS 23 (IBM). Unless otherwise indicated, data are presented as mean ± SD. *P* values less than 0.05 were considered statistically significant.

### Study approval.

Written informed consent was acquired for all patient samples, and animal studies were performed according to University of South Florida IACUC–approved protocols.

## Author contributions

AFM, AFL, and KLM wrote the manuscript. AFM, HH, BSM, NBL, GAW, ALA, AV, LZ, NDV, and KLM performed experiments and analyzed data. MAR, EP, DAS, and OAW analyzed data.

## Supplementary Material

Supplemental data

## Version 1 07/05/2022

In-Press Preview

## Version 2 08/08/2022

Electronic publication
