# Supplementary material for: Somatic gene mutations expose cytoplasmic DNA to co-opt the cGAS/STING/NLRP3 axis in myelodysplastic syndromes
Source: JCI Insight. 2022 Aug 8;7(15):e159430. doi: 10.1172/jci.insight.159430 (PMC9462508; doi:10.1172/jci.insight.159430)
Supplement: Supplemental data [file jciinsight-7-159430-s083.pdf]

**Somatic Gene Mutations Expose Cytoplasmic DNA to Co-Opt the cGAS-STING-NLRP3 Axis in Myelodysplastic Syndromes**

Amy F. McLemore[1], Hsin-An Hou[2], Benjamin S. Meyer[1], Nghi Lam[1], Grace A. Ward[3], Amy L. Aldrich[1], Matthew A. Rodrigues[4], Alexis Vedder[1], Ling Zhang[5], Eric Padron[1], Nicole D. Vincelette[1], David A. Sallman[1], Omar Abdel-Wahab[6], Alan F. List[1,8] and Kathy McGraw[1],[7]

1. Department of Malignant Hematology, Moffitt Cancer Center & Research institute, Tampa, FL
2. Department of Internal Medicine, National Taiwan University Hospital (NTUH), Taipei, Taiwan
3. Cancer Biology Ph.D. Department, University of South Florida, Tampa, FL
4. Luminex Corporation, Seattle, WA
5. Department of Hematopathology, Moffitt Cancer Center & Research institute, Tampa FL
6. Human Oncology and Pathogenesis Program and Leukemia Service, Memorial Sloan Kettering Cancer Center, New York, NY
7. Precision Biosciences, Durham, NC 27701
8. Laboratory of Receptor Biology and Gene Expression, National Cancer Institute, National Institutes of Health, Bethesda, Maryland 20892, USA;

**Supplemental Material**

1. Supplemental Figure
2. Supplemental Tables

**Supplemental Figure S1. Immortalized cell line validation.** (A) Dot blot of 5-hydroxymethylcytosine in *Tet2*$^{-/-}$ and wildtype (Bl/6) immortalized cell lines. Propidium iodide (PI) is used as a loading control. (B) Sequence of *Srsf2* $^{P95H}$ and wild type (MX1-Cre) immortalized cells with the wild type reference sequence demonstrating mutation.

A.

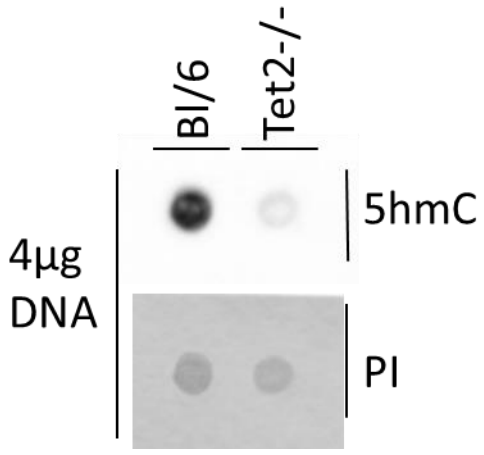


B.

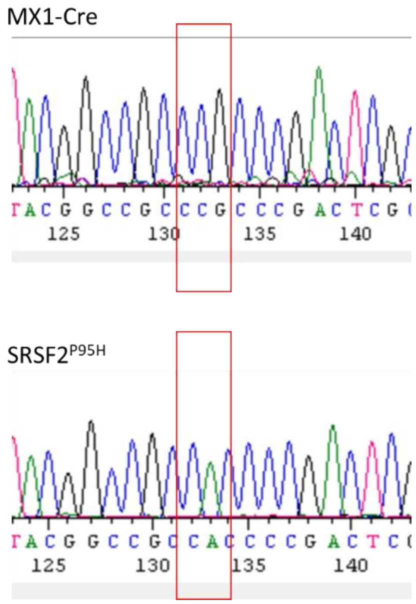


## Supplemental Tables:

**Supplemental Table S1. RNA polymerase expression in MDS.** Gene expression of all subunits for POLR1, POLR2, and POLR3 were significantly upregulated in MDS BM-MNCs (n = 213) compared to age-matched donors (n = 20).

| | | |
|---|---|---|
| Homo sapiens polymerase (RNA) I polypeptide A, 194kDa (POLR1A), mRNA. | POLR1A | P < 0.0001 |
| Homo sapiens polymerase (RNA) I polypeptide B, 128kDa (POLR1B), transcript variant 2, mRNA. | POLR1B | P < 0.0001 |
| Homo sapiens polymerase (RNA) I polypeptide C, 30kDa (POLR1C), mRNA. | POLR1C | P < 0.0001 |
| Homo sapiens polymerase (RNA) I polypeptide D, 16kDa (POLR1D), transcript variant 3, mRNA. | POLR1D | P < 0.0001 |
| Homo sapiens polymerase (RNA) I polypeptide E, 53kDa (POLR1E), transcript variant 1, mRNA. | POLR1E | P < 0.0001 |
| Homo sapiens polymerase (RNA) II (DNA directed) polypeptide A, 220kDa (POLR2A), mRNA. | POLR2A | P = 0.0005 |
| Homo sapiens polymerase (RNA) II (DNA directed) polypeptide B, 140kDa (POLR2B), mRNA. | POLR2B | P < 0.0001 |
| Homo sapiens polymerase (RNA) II (DNA directed) polypeptide C, 33kDa (POLR2C), mRNA. | POLR2C | P < 0.0001 |
| Homo sapiens polymerase (RNA) II (DNA directed) polypeptide D (POLR2D), mRNA. | POLR2D | P < 0.0001 |
| Homo sapiens polymerase (RNA) II (DNA directed) polypeptide E, 25kDa (POLR2E), mRNA. | POLR2E | P = 0.0004 |
| Homo sapiens polymerase (RNA) II (DNA directed) polypeptide F (POLR2F), mRNA. | POLR2F | P < 0.0001 |
| Homo sapiens polymerase (RNA) II (DNA directed) polypeptide G (POLR2G), mRNA. | POLR2G | P < 0.0001 |
| Homo sapiens polymerase (RNA) II (DNA directed) polypeptide H (POLR2H), transcript variant 2, mRNA. | POLR2H | P < 0.0001 |
| Homo sapiens polymerase (RNA) II (DNA directed) polypeptide I, 14.5kDa (POLR2I), mRNA. | POLR2I | P < 0.0001 |
| Homo sapiens polymerase (RNA) II (DNA directed) polypeptide J, 13.3kDa (POLR2J), mRNA. | POLR2J | P < 0.0001 |
| Homo sapiens polymerase (RNA) II (DNA directed) polypeptide K, 7.0kDa (POLR2K), mRNA. | POLR2K | P < 0.0001 |
| Homo sapiens polymerase (RNA) II (DNA directed) polypeptide L, 7.6kDa (POLR2L), mRNA. | POLR2L | P = 0.0297 |
| Homo sapiens polymerase (RNA) II (DNA directed) polypeptide J3 (POLR2J3), mRNA. | POLR2J3 | P = 0.4111 |

| | | |
|---|---|---|
| Homo sapiens polymerase (RNA) III (DNA directed) polypeptide A, 155kDa (POLR3A), mRNA. | POLR3A | P < 0.0001 |
| Homo sapiens polymerase (RNA) III (DNA directed) polypeptide B (POLR3B), transcript variant 2, mRNA. | POLR3B | P < 0.0001 |
| Homo sapiens polymerase (RNA) III (DNA directed) polypeptide C (62kD) (POLR3C), mRNA. | POLR3C | P < 0.0001 |
| Homo sapiens polymerase (RNA) III (DNA directed) polypeptide D, 44kDa (POLR3D), mRNA. | POLR3D | P < 0.0001 |
| Homo sapiens polymerase (RNA) III (DNA directed) polypeptide E (80kD) (POLR3E), transcript variant 1, mRNA. | POLR3E | P < 0.0001 |
| Homo sapiens polymerase (RNA) III (DNA directed) polypeptide F, 39 kDa (POLR3F), transcript variant 1, mRNA. | POLR3F | P < 0.0001 |
| Homo sapiens polymerase (RNA) III (DNA directed) polypeptide G (32kD) (POLR3G), mRNA. | POLR3G | P < 0.0001 |
| Homo sapiens polymerase (RNA) III (DNA directed) polypeptide G (32kD)-like (POLR3GL), mRNA. | POLR3GL | P < 0.0001 |
| Homo sapiens polymerase (RNA) III (DNA directed) polypeptide H (22.9kD) (POLR3H), transcript variant 3, mRNA. | POLR3H | P < 0.0001 |
| Homo sapiens polymerase (RNA) III (DNA directed) polypeptide K, 12.3 kDa (POLR3K), mRNA. | POLR3K | P < 0.0001 |
| Homo sapiens polymerase (RNA) mitochondrial (DNA directed) (POLRMT), mRNA. | POLRMT | P < 0.0001 |

**Table S2. PCR and CRISPR Primers.**

| Gene | Forward Primer | Reverse Primer |
|---|---|---|
| *ISG15* | GAGAGGCAGCGAACTCATCT | CTTCAGCTCTGACACCGACA |
| *CXCL10* | AAGTGGCATTCAAGGAGTACCT | AACACGTGGACAAAATTGGCT |
| *SAMD9L* | AGGAAAATCCTGCATTTCCAGAG | GCAAGGGGCTTACACTTTCC |
| *GAPDH* | GAAGGTGAAGGTCGGACT | GAAGATGGTGATGGGATTTC |
| *Isg15* | TGACGCAGACTGTAGACACG | TGGGGCTTTAGGCCATACTC |
| *Cxcl10* | GACGGTCCGCTGCAACTG | CTTCCCTATGGCCCTCATTCT |
| *Ccl5* | CCCTCACCATCATCCTCACT | AGAGGTAGGCAAAGCAGCAG |
| *Gapdh* | GAAGGTGAAGGTCGGACT | GAAGATGGTGATGGGATTTC |
| **CRISPR Guide** | **Forward Primer** | **Reverse Primer** |
| cGAS_sg3 | TGTTTAAACTGGAAGTCCCC | GGGGACTTCCAGTTTAAACA |
| cGAS_sg4 | AAATTCAAAAGAATTCCACG | CGTGGAATTCTTTTGAATTT |
| Scrambled | CACCGACGGAGGCTAAGCGTCGCA | TGCGACGCTTAGCCTCCGTCGGTG |

**Table S3. Antibodies.**

| Antibody | Manufacturer | Catalog Number | Dilution |
|---|---|---|---|
| S9.6 APC | Absolute Antibody, Wilton, UK | Ab01137-24.1 | 1:500 |
| Lamin B1 | Invitrogen, Carlsbad, CA | MA1-06103 | 1:1000 |
| cGAS FITC | LifeSpan Biosciences, Seattle, WA | LS-C247018 | 1:500 |
| P-IRF3 PE | Cell Signaling Technology, Danvers, MA | 83611 | 1:50 |
| CD71 AF700 | BD Biosciences, Franklin Lakes, NJ | 563769 | 1:100 |
| ASC AF647 | Santa Cruz Biotechnology, Santa Cruz, CA | sc-514414 AF647 | 1:500 |
| IL-1β PerCP | R&D Systems, Minneapolis, MN | IC201C | 1:500 |
| Caspase-1 | Cell Signaling Technology, Danvers, MA | 2225 | 1:1000 |
| Beta Actin | Sigma, St. Louis, MO | A5316 | 1:1000 |
| IRF3 | Cell Signaling Technology, Danvers, MA | 4302 | 1:1000 |
| Histone H3 | Cell Signaling Technology, Danvers, MA | 4499 | 1:1000 |
